# Systematic characterization of extracellular vesicles from potato (*Solanum tuberosum* cv. Laura) roots and peels: biophysical properties and proteomic profiling

**DOI:** 10.3389/fpls.2024.1477614

**Published:** 2024-11-15

**Authors:** Gayandi Ekanayake, Johanna Piibor, Getnet Midekessa, Kasun Godakumara, Keerthie Dissanayake, Aneta Andronowska, Rajeev Bhat, Alireza Fazeli

**Affiliations:** ^1^ Institute of Veterinary Medicine and Animal Sciences, Estonian University of Life Sciences, Tartu, Estonia; ^2^ Department of Pathophysiology, Institute of Biomedicine and Translational Medicine, University of Tartu, Tartu, Estonia; ^3^ Department of Anatomy, Faculty of Medicine, University of Peradeniya, Peradeniya, Sri Lanka; ^4^ Institute of Animal Reproduction and Food Research, Polish Academy of Sciences, Olsztyn, Poland; ^5^ ERA-Chair for Food (By-) Products Valorisation Technologies (VALORTECH), Estonian University of Life Sciences, Tartu, Estonia; ^6^ Division of Clinical Medicine, School of Medicine and Population Health, University of Sheffield, Sheffield, United Kingdom

**Keywords:** *Solanum tuberosum*, plant extracellular vesicles, agricultural waste, value addition and sustainability, proteomics, plant biotic and abiotic stress, valorization

## Abstract

**Introduction:**

Extracellular vesicles (EVs) facilitate inter and intra-species/kingdom communication through biomolecule transfer, including proteins and small RNAs. Plant-derived EVs, a hot topic in the field, hold immense capability both as a potential biomarker to study plant physiology and as a biomaterial that can be mass-produced to be used in various industries ranging from cosmetics and food additives to biological pesticides. However, a systematic characterization of plant EVs is required to establish a foundation for further applications and studies.

**Methods:**

In this study, EVs were enriched from hydroponically cultivated potato plants (*Solanum tuberosum*, cv. Laura). We isolated EVs from root exudates and the apoplastic wash of potato peels using vacuum infiltration. These EVs were then systematically characterized for their biophysical and chemical properties to compare with standard EV characteristics and to explore their roles in plant physiology.

**Results:**

Biophysical and chemical analyses revealed morphological similarities between potato root and peel-derived EVs. The average diameter of root-derived EVs (164.6 ± 7.3 nm) was significantly larger than that of peel-derived EVs (132.2 ± 2.0 nm, *p* < 0.004). Liquid chromatography–mass spectrometry (LC-MS) demonstrated substantial protein enrichment in purified EVs compared to crude samples, with a 42% enrichment for root EVs and 25% for peel EVs. Only 11.8% of the identified proteins were common between root and peel EVs, with just 2% of significantly enriched proteins shared. Enriched pathways in both EV proteomes were associated with responses to biotic and abiotic stress, suggesting a defensive role of EVs in plants.

**Discussion:**

With further experimentation to elucidate the specific methods of communication, these findings increase the details known about plant EVs in terms of their physical and chemical characteristics and their potential functions, aiding in sustainable agricultural waste utilization for large-scale EV production, aligning with the concept of “valorization”.

## 1 Introduction

In multicellular organisms, a variety of vesicles enclosed by biological membranes are present, both intracellularly and extracellularly. These include endosomes and lysosomes within cells and extracellular vesicles (EVs) in the extracellular space. These EVs possess the capability to encapsulate, transport, or store biomolecules, including proteins, bioactive lipids, miRNAs, and other metabolites (Di Bella, 2022; Liu and Wang, 2023). Recent findings have emphasized the role of extracellular vesicles in facilitating intercellular communication in various physiological and pathological processes (Dolcetti et al., 2020; Busatto et al., 2021; Papareddy et al., 2024) by delivering their cargo/message to recipient cells through targeted interactions (Colombo et al., 2014; Pucci and Raimondo, 2021). Over the past two decades, scientists have observed that plant cells secrete vesicles that are of equivalent quality in terms of both morphology and biological function to mammalian cell-derived EVs (An et al., 2007).

Although the initial records of small EVs from the plant apoplast (the plant extracellular fluid) date back to as early as the 1960s (Halperin and Jensen, 1967), the field of study was largely ignored until the last decade. This lack of attention was due to uncertainty within the scientific community about the potential existence of EVs in plants, primarily due to the presence of the plant cell wall. However, through the utilization of advanced molecular, cellular, and proteomic techniques, researchers have managed to collect a plethora of evidence to confirm that plants do indeed produce various types of vesicles that play roles in responses to both biotic and abiotic stresses. Furthermore, these vesicles have been shown to participate in defense mechanisms against pathogens (Hansen and Nielsen, 2017; Rutter and Innes, 2017) and to facilitate the transport of mRNAs, miRNAs, bioactive lipids, and proteins not only between plant cells but also into animal, fungal, and bacterial cells, enabling inter-kingdom communications. Cumulatively, research also supports that the diverse bioactive molecules contained in plant EVs have the potential to contribute to enhancing different aspects of animal and human health including theragnostics, nutritional support, and cosmetics (Reiner and Somoza, 2019; Andrews et al., 2021; Lo et al., 2024). Furthermore, early records on the defensive role of plant EVs indicate their potential use in crop protection and horticulture (Andreu and Yáñez-Mó, 2014; Liu et al., 2021); by managing pathogens and improving crop resilience, plant EVs could revolutionize sustainable farming practices and contribute significantly to global food security.

Despite the growing interest in plant-based EVs, the enrichment and purification of EVs from plant tissues remain a significant challenge. This challenge arises from the lack of a simple and widely applicable method for isolation, purification, and characterization. According to reports from the International Society for Extracellular Vesicles (ISEV), only a minority of researchers have successfully purified plant EVs from extracellular apoplastic fluids without causing cell or tissue rupture, which can lead to increased intercellular contamination (Ju et al., 2013; Yang et al., 2018; Pinedo et al., 2021). Existing techniques for sample preparation and isolation from plants can be broadly classified into disruptive and non-disruptive methods. Disruptive methods primarily involve soft tissue disruption, employing one or several physical techniques such as grinding, squeezing, or blending. These methods can yield a large number of vesicular fractions, but with the risk of causing cell or tissue rupture, leading to potential intercellular contamination (Wang et al., 2013; Pocsfalvi et al., 2018). At present, the generic term “plant-derived nanovesicles (PDNVs)” has been established to distinguish these vesicular fractions (Pinedo et al., 2021). In contrast, vesicles obtained through non-disruptive methods, such as natural release via roots or vacuum infiltration for apoplastic fluid extraction, are referred to as plant extracellular vesicles (plant EVs) (Rutter and Innes, 2017; De Palma et al., 2020; Boccia et al., 2022). These methods, while potentially yielding fewer EVs, reduce the risk of intercellular contamination by avoiding tissue disruption. However, a universally accepted “gold standard” separation method has not yet been established, indicating the need for further research in this area.

To date, no biomarkers specific to plant EVs have been established to differentiate physiological or pathological states in any plant species. Although not thoroughly explored, research by Yang et al. (2018) suggests the possibility of distinct contents in vesicles obtained from different parts of the same plant (Yang et al., 2018). It is hypothesized that factors influencing the chemical makeup of plants could subsequently impact the composition of both plant-derived vesicles (PDVs) and plant EVs (Nemati et al., 2022). Comprehensive analyses of plant EV content have been conducted for only a limited number of plant species (Pinedo et al., 2021), and potato is not among this number, despite being a vastly consumed major crop plant. Before attempting to elucidate the function of EVs in potato roots and peels, it is imperative to conduct a systematic characterization of the particles. There is a need to encourage further research to comprehensively grasp the biologically active components conveyed by plant EVs, thus unlocking their potential in theragnostic applications (Ju et al., 2013; Yang et al., 2018; Pinedo et al., 2021).

Understanding the composition of potato root- and peel-derived EVs will be a major step forward in understanding their potential function in the physiology of the plant, such as the role they play in defending the plant against pathogens. Once sufficiently understood, these physiological functions can be further improved using breeding technologies to produce more robust and resistant crops, decreasing the use of chemical pesticides. Once their function and method of action are deciphered, EVs themselves could potentially be used as fertilizers or organic pesticides.

The primary objective of the current study was to explore potential differences in the characteristics and composition of EVs originating from sustainable sources such as potato (*Solanum tuberosum* cv. Laura) peels and roots, aiming to understand the significant variations that may exist in EVs from distinct parts of a plant. Such insights could lead to the identification of distinctive markers that differentiate these EVs and further unravel their functions in plant physiology employing bioinformatic technology.

## 2 Materials and methods

### 2.1 Potato plant growth, collection of root exudates, and root growth medium

Commercially available healthy potato tubers (*S. tuberosum* cv. Laura) in the size B (5–6 cm in diameter) category were purchased from a local vegetable supplier (Laheotsa OÜ, Harjumaa, Estonia). Three separate batches of tubers were used as biological replicates. The surface of these selected tubers was disinfected by keeping them immersed in a sodium hypochlorite solution (1% v/w, household bleach solution) for 4–5 min to eliminate any surface contaminants. Following disinfection, the tubers were washed with distilled water with two to three drops (approximately 150 µL) of Tween 20 (Sigma-Aldrich, Taufkirchen, Germany) to enhance the removal of any residual contaminants. The tubers were further surface-sterilized using 70% ethanol for 1–2 min where ethanol (70%) was expected to act as a broad-spectrum antimicrobial agent. To conclude the surface-sterilization process, the tubers were washed three times with ultrapure Milli-Q^®^ water (08.2205, TKA Wasseraufbereitungssysteme GmbH, Niederelbert, Germany). The final wash was carried out in order to ensure the removal of any residual ethanol or Tween 20, leaving the potato tubers thoroughly cleaned and surface-sterilized, ready for the next steps in the experiment (Figueiredo et al., 2022). Subsequently, the tubers were allowed to sprout until approximately 2-cm-long sprouts emerged from the surface. The sprouted tubers were immersed in a nutrient solution containing essential macro elements (nitrogen, phosphorus, and potassium) and vital micronutrients (calcium and magnesium) for a duration of 2–3 weeks. A deep-water culture-based hydroponic system (VEVOR 4 Buckets DWC, ASIN: B09R1P9GV2; Vevor, Shanghai, China), situated in a walk-in chamber, facilitated further root development. The nutrient solution composition included calcium nitrate (CaNO_3_) at 44.65 mg/L, potassium nitrate (KNO_3_) at 98.35 mg/L, monopotassium phosphate (KH_2_PO_4_) at 37.4 mg/L, and magnesium sulfate (MgSO_4_) at 25 mg/L. The pH range of the growth solution was carefully maintained at 6.0 ± 0.2 (Tessema et al., 2017). The conditions in the chamber were maintained as follows: a temperature of 20°C ± 2°C (day and night), 95% relative humidity, and a 9:15 h light:dark photoperiod. Daylight was supplemented using a grow light system (MARS HYDRO 2024 New TS1000 150W LED Grow Light; Mars Hydro, Shenzhen, CHINA) with a photosynthetic photon flux density (PPFD) of 200 μmol m^−2^ s^−1^. The nutrient solution was replenished weekly. After 3 weeks, according to the procedure described by De Palma et al. (2020), the tubers were removed from the nutrient solution, and the roots were washed meticulously two to three times using ultrapure Milli-Q^®^ water (08.2205, TKA Wasseraufbereitungssysteme GmbH, Niederelbert, Germany) (De Palma et al., 2020). The nutrient solution in the hydroponic system was replaced with 1,000 mL of ultrapure Milli-Q^®^ water (08.2205, TKA Wasseraufbereitungssysteme GmbH, Niederelbert, Germany), and the tubers were transferred back to the system. Further growth of the tubers was sustained for another 48 h at the previously mentioned conditions. Subsequently, the remaining root growth medium (water sampling solution, approximately 500 mL) was collected using a 50-mL sterile serological pipette and transferred to a 500-mL sterile Pyrex glass bottle. This collected sample was subsequently used for EV isolation. In total, three independent experiments were conducted, each involving the establishment of separate hydroponic systems to cultivate potatoes under identical conditions. From each of these systems, samples were collected for both particle isolation and analysis. Each hydroponic setup contained four to five potatoes (approximately 1 kg of total weight). These potatoes were chosen to be of similar size and weight across all setups.

### 2.2 Extraction of apoplastic fluid from potato peel

The surface of selected potato tubers was disinfected, washed, and surface-sterilized following the method described by Figueiredo et al. (2022). Potatoes (≈ 2 kg per replicate) were evenly peeled using a sterilized manual potato peeler with a straight blade to avoid the flesh. The extraction fluid was collected following the established protocols based on a vacuum infiltration-centrifugation procedure with few alterations (Regente et al., 2009). Briefly, the potato peels were submerged in a vesicle isolation buffer (VIB; 20 mM MES, 2 mM CaCl_2_, and 0.1 M NaCl, pH 6) and subjected to a vacuum pulse of 30 Pa for 10 min in a vacuum chamber (BACOENG 3 Vacuum degassing chamber and pump kit, P0774; BACO Engineering, Suzhou, China). Following this process, the infiltrated peels were recovered, dried on a filter paper to remove excess buffer solution, and then positioned upright in 20-mL syringes. All steps were carried out on an open laboratory bench at room temperature (25°C ± 2°C). Subsequently, the peels were placed in 50-mL Falcon tubes and centrifuged for 20 min at 1,000 ×*g* at 4°C. The resulting extraction fluid was recovered. A total of three distinct sample collections were conducted, each comprising three technical replicates. For each experiment, 2 kg of potatoes were used.

### 2.3 Isolation and purification of potato-derived EVs

Every effort was taken to follow the standard guidelines set by the ISEV in the Minimal information for studies of extracellular vesicles (MISEV2023) in enriching and characterizing EVs in the current study (Welsh et al., 2024).

Before initiating EV isolation, a volume of 1.0–2.0 mL from each replicate of unprocessed crude samples of peel apoplastic extraction fluid and root growth medium was separated and stored at −80°C for future proteomic analysis, along with the processed and enriched EV samples.

The isolation of EVs from the collected peel apoplastic extraction fluid and root growth medium was performed according to the protocol published by Hasan et al. (2021) with slight modifications (Hasan et al., 2021). Briefly, the collected apoplastic fluid was centrifuged at 500 ×*g* for 10 min at 4°C. The resulting supernatant was again centrifuged at 3,000 ×*g* for 15 min at 4°C, and thereafter, the final supernatant was centrifuged at 10,000 ×*g* for 15 min at 4°C. This series of centrifugation steps was aimed at eliminating cells, cell debris, and other impurities present in the samples. To isolate EVs, the final supernatant was retrieved and concentrated to 500 µL using Amicon^®^ Ultra-15 centrifugal filter units (10 kDa cut-off, Merck Millipore Ltd., Dublin, Ireland) by centrifuging at 3000 ×*g* for 20–30 min at 4°C. EVs were isolated from concentrated sample solutions using size exclusion chromatography (SEC) (Econo-pac^®^ Disposable chromatography column, Bio-Rad, Berkeley, CA, USA) packed with a 10-cm column of cross-linked 4% agarose matrix with 90-µm beads (Sepharose 4 fast flow™, GE HealthCare Bio-Sciences AB, Uppsala, Sweden) and using Dulbecco’s phosphate-buffered saline (DPBS; Sigma-Aldrich, St. Louis, MO, USA) as the equilibration buffer. To determine the characteristics of EV enrichment, 20 fractions of 500 µL were collected from each of the concentrated potato root and peel samples.

### 2.4 Identification of EV fractions

#### 2.4.1 Nanoparticle tracking analysis

The size and concentration of particles present in each fraction were determined using Nanoparticle Tracking Analysis (NTA) ZetaView^®^ (PMX 110 v3.0 instrument from particle Metrix GmbH, Inning am Ammersee, Germany). The data were analyzed using the ZetaView NTA software (Dissanayake et al., 2021; Midekessa et al., 2021). Calibration of the instrument was conducted following the instructions provided by the manufacturer with a known concentration of 100-nm polystyrene nanoparticles (Applied Microspheres BV, Leusden, The Netherlands; Catalog no. 10100). The particle concentration and size distribution were measured with the following scatter settings: sensitivity at 85, shutter value at 70, frame rate at 30 frames per second (fps), and frames at 11 frames per cycle. Each sample was measured in triplicate at 25°C. To minimize inter-sample contamination, the cell chamber of the instrument was washed between samples with Milli-Q^®^ followed by DPBS (Sigma-Aldrich, USA). Depending on the nanoparticle size ranges and concentrations, fractions of interest in each potato root and peel EV sample were merged per sample. The respective pooled EV fractions were further concentrated to 250 µL using Amicon^®^ Ultra-2 mL centrifugal filters (Merck Millipore Ltd., Darmstadt, Germany, 10 kDa cut-off) at 3,000 ×*g*. Concentrated and purified EV samples were stored at −80°C until further characterization.

#### 2.4.2 Protein content determination

The protein concentrations of the collected fractions were determined using the Bradford assay (Bradford, 1976; Webber and Clayton, 2013). Initially, a dilution series of bovine serum albumin (BSA) standard solution (2 mg/mL, Sigma-Aldrich, USA) was prepared (1.4 mg/mL, 1.0 mg/mL, 0.5 mg/mL, 0.25 mg/mL, and 0.125 mg/mL) along with DPBS as a negative control. Triplicates of each test sample, standard, and negative control (5 μL per well) were placed in a 96-well microplate. A volume of 95 μL of Bradford reagent (Sigma-Aldrich, St. Louis, MO, USA) was added to each well. The microplate was covered with aluminum foil and placed on a shaker for 30 s for homogenous mixing. Then, the plate was incubated at room temperature for 15–30 min followed by measurement of absorbance of both the standard and the samples using a spectrophotometer (Ledetect96 Microplate Reader; Biomed Dr. Wieser GmbH, Salzburg, Austria) at a 620-nm wavelength. The protein concentration of each fraction was calculated separately.

### 2.5 Transmission electron microscopy

Purified potato peel and root EV samples (20 µL) were placed on formvar carbon-coated 200 mesh copper grids (Agar Scientific, Stansted, UK) for 20 min. Then, the grids were incubated with 2% uranyl acetate (Polysciences, Warrington, PA, USA) for 2 min to obtain contrasted images of the EVs. Potato peel and root EVs were visualized using JEM 1400TEM (JEOL Ltd., Tokyo, Japan; with Morada TEM CCD camera, Olympus, Hamburg, Germany) at 80 kV. The digital images of EVs were captured using a numerical camera (Morada TEM CCD camera, Olympus, Hamburg, Germany).

The areas imaged by transmission electron microscopy (TEM) were selected based on the presence of characteristic EV morphology. Initially, the sample grids were scanned at low magnification (approximately ×1,000 to ×10,000) to identify regions with a high density of vesicles. Once potential areas were identified, higher magnification (approximately ×25,000 to ×50,000) was used to capture detailed images of individual vesicles. The selection criteria included the size, shape, and uniformity of the vesicles, ensuring that the imaged areas were representative of the overall sample.

### 2.6 Mass spectrometry and proteomic data analysis

#### 2.6.1 Sample preparation for LC-MS/MS

All samples were processed in a randomized order. Proteins were precipitated by adding precipitation solution (sodium deoxycholate 4 mg/mL in 100% Trichloroacetic acid (TCA)) to 20% (v/v), incubated overnight at 4°C, and then pelleted by centrifugation at 17,000 ×*g* for 15 min. Sodium deoxycholate, a detergent that solubilizes membrane proteins, was used in the precipitation solution. The pellets were washed twice with acetone, pelleted by centrifugation again, and then air-dried. Rough protein concentrations were estimated by Micro BCA assay (Thermo Fisher Scientific, Waltham, MA, USA). The precipitated proteins were then solubilized in 7 M urea, 2 M thiourea, and 100 mM ammonium bicarbonate buffer (ABC); reduced with 5 mM dithiothreitol for 1 h; and alkylated with 20 mM chloroacetamide in the dark for 1 h. Pre-digestion with 1:50 (enzyme to protein ratio) Lys-C (Fujifilm Wako Pure Chemical, Osaka, Japan) was carried out for 4 h at 25°C. Next, the solution was diluted five times with 100 mM ABC, and further digestion with 1:50 dimethylated trypsin (Sigma-Aldrich) was carried out overnight at 25°C. Samples were then acidified with trifluoroacetic acid (TFA) to 1.0% and desalted on in-house made C18 material (3M) solid phase extraction tips. Purified peptides were reconstituted in 0.5% TFA for nano-liquid chromatography–tandem mass spectrometry (nano-LC-MS/MS).

#### 2.6.2 LC-MS/MS analysis

To determine the final peptide injection amounts, a 20-fold dilution of the final sample was first pre-analyzed by LC-MS/MS, integrating the peptide signal from each sample, and then an equal amount of each sample was injected for a final run. LC-MS/MS analysis was carried out by loading injected peptides onto a 0.3 × 5 mm trap-column (5 µm C18 particles, Dionex) using an Ultimate 3000 RSLCnano system (Dionex, Sunnyvale, CA, USA). Peptides were eluted onto an in-house packed (3 µm C18 particles, Dr Maisch, Ammerbuch, Germany) analytical 50 cm × 75 µm emitter-column (New Objective, Littleton, MA, USA) and separated with an A to B 8%–40% 30-min gradient (buffer A, 0.1% formic acid; buffer B, 80% acetonitrile + 0.1% formic acid). Separated peptides were sprayed into a Q Exactive HF mass spectrometer (Thermo Fisher Scientific) via a nano-electrospray source (positive mode, spray voltage of 2.5 kV). The MS was operated with a top-12 data-dependent acquisition strategy. Briefly, one 350–1,400 *m*/*z* full MS scan at a resolution setting of R = 60,000 at 200 *m*/*z* was followed by higher-energy collisional dissociation fragmentation (normalized collision energy of 26) of the 12 most intense ions (z: +2 to +6) at R = 30,000. MS and MS/MS ion target values were 3,000,000 and 100,000 ions with 50- and 45-ms injection times, respectively. MS/MS isolation was carried out with 1.2 *m*/*z* isolation windows. Dynamic exclusion was limited to 20 s.

#### 2.6.3 Raw data analysis

MS raw files were processed with the MaxQuant software package (version 2.0.3.0) (Cox and Mann, 2008). Methionine oxidation and protein N-terminal acetylation were set as variable modifications, while cysteine carbamidomethylation was defined as a fixed modification. The search was performed against the UniProt (www.uniprot.org) *S. tuberosum* proteome database (downloaded: June 2023) using the tryptic digestion rule (cleavages after lysine and arginine without proline restriction). Only identifications with at least one peptide ≥ 7 amino acids long (with up to two missed cleavages) were accepted, and transfer of identifications between runs based on accurate mass and retention time was allowed. Label-free normalization with the MaxQuant LFQ algorithm was also applied. The LFQ ratio count (i.e., the number of quantified peptides to report a protein intensity) was set to two. Peptide-spectrum match and protein false discovery rate (FDR) were kept below 1% using a target-decoy approach. All other parameters were set by default. The resulting raw data files of proteins and peptides are deposited in the Figshare repository under https://doi.org/10.6084/m9.figshare.25607601.v1.

### 2.7 Bioinformatic analysis

#### 2.7.1 Differential enrichment analysis of proteomes and network analysis

The R package Differential Enrichment analysis of Proteomics data (DEP) (Zhang et al., 2018) was used for the differential enrichment analysis of EVs and crude proteomes in both potato roots and peels. For further analysis, the log2-transformed LFQ intensity values were used for proteins detected in at least two out of three replicates of either crude or EV protein samples. The root and peel samples were separately analyzed as the EV proteome against the crude proteomes. A total of 858 proteins were selected for further analysis in the potato peel proteome, while 206 proteins were chosen for analysis in the potato root proteome. A qualitative comparison was made to identify any shared EV proteins between crude and purified samples. In the analysis, the k-nearest neighbor (knn) approach was used for data imputation. The cut-off values of the significantly enriched proteins were adjusted to a *p*-value of 0.05 (the Benjamini–Hochberg method) and a log2 fold change of ±1.5. The resulting list of differentially expressed proteins was then utilized for network analysis.

Network analysis was performed on differentially expressed proteins using the “enricher ()” function of the cluster Profiler package (Yu et al., 2012) to identify overrepresented Gene Ontology (GO) terms. GO term annotations for *S. tuberosum* proteins were obtained from Ensembl plant Biomart using the biomaRt package (Durinck et al., 2009). The whole proteome of *S. tuberosum* was used as the background “universe” for the network analysis.

#### 2.7.2 Identifying EV-specific markers in potato EV proteomes

Recently published articles attempting to compile lists of probable universal plant EV markers were used to build a database of probable universal plant EV markers (Oshikawa et al., 2016; Regente et al., 2017; Rutter and Innes, 2017, 2020; Movahed et al., 2019; Timms et al., 2019; Woith and Melzig, 2019; Woith et al., 2019; De Palma et al., 2020; He et al., 2021; Woith et al., 2021; Yugay et al., 2023; Saito et al., 2024). The 30 proteins most frequently mentioned in the selected literature were used for further analysis.

Proteins identified in each sample (potato root-derived EVs and potato peel-derived EVs) were explored for matching proteins compared to the EV marker database. In cases where published EV marker proteins were not annotated in the *S. tuberosum* proteome, the nearest homologs (bitscore > 100, E-value < 0.0001) were identified using the BLASTp algorithm, and the sample proteomes were explored for the presence of the homologs.

## 3 Results

**Figure 1:**
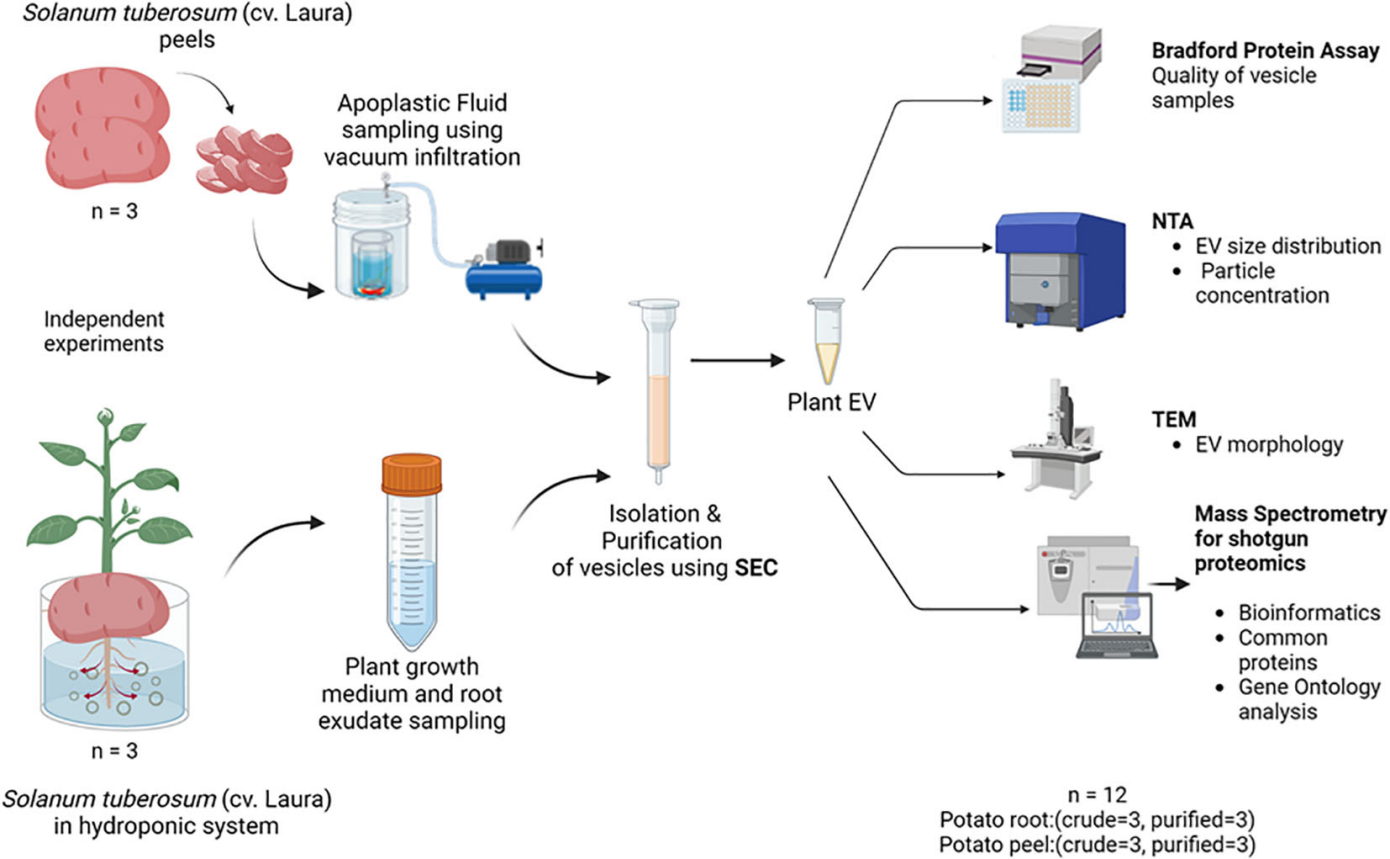
The experimental design used for the collection, isolation, and characterization of extracellular vesicles released by potato (cv. Laura) roots and peels. Independent experiments were carried out to collect initial sampling solutions containing root exudates of potato (*Solanum tuberosum*, cultivar: Laura) plants grown hydroponically and apoplastic washes collected from potato peels of the same cultivar by a vacuum infiltration method using a vesicle isolation buffer. Three replicates were conducted for each experiment. The purification process was a combination of differential centrifugation steps and size exclusion chromatography (SEC), where 20 purified fractions (500 µL each) of the samples were collected for further examination. Biophysical and chemical analyses were conducted on these extracellular vesicles (EVs) using the Bradford protein assay, nanoparticle tracking analysis (NTA), and transmission electron microscopy (TEM). The EV proteome of the samples was evaluated using mass spectrometry where purified EV samples (n = 3) and crude samples (n = 3) from each potato peel and root sample were used for comparison.

The study comprised two main experiments (**Figure 1**). First, potato root- and peel-derived EVs were characterized using NTA to determine particle size and concentration, followed by a Bradford protein assay for protein content. Fractions with the highest particle abundance were imaged via TEM to confirm EV morphology, with all experiments performed in triplicate. Second, quantitative LC-MS/MS proteomics was used to analyze EV protein content, and differential enrichment analysis was conducted to compare protein abundance in crude and purified EV samples (n = 3). To identify EV-specific markers in potato EV proteomes, a database of probable universal plant EV markers was compiled from recent literature.

### 3.1 Isolation and characterization of potato peel- and root-derived EVs

**Figure 2:**
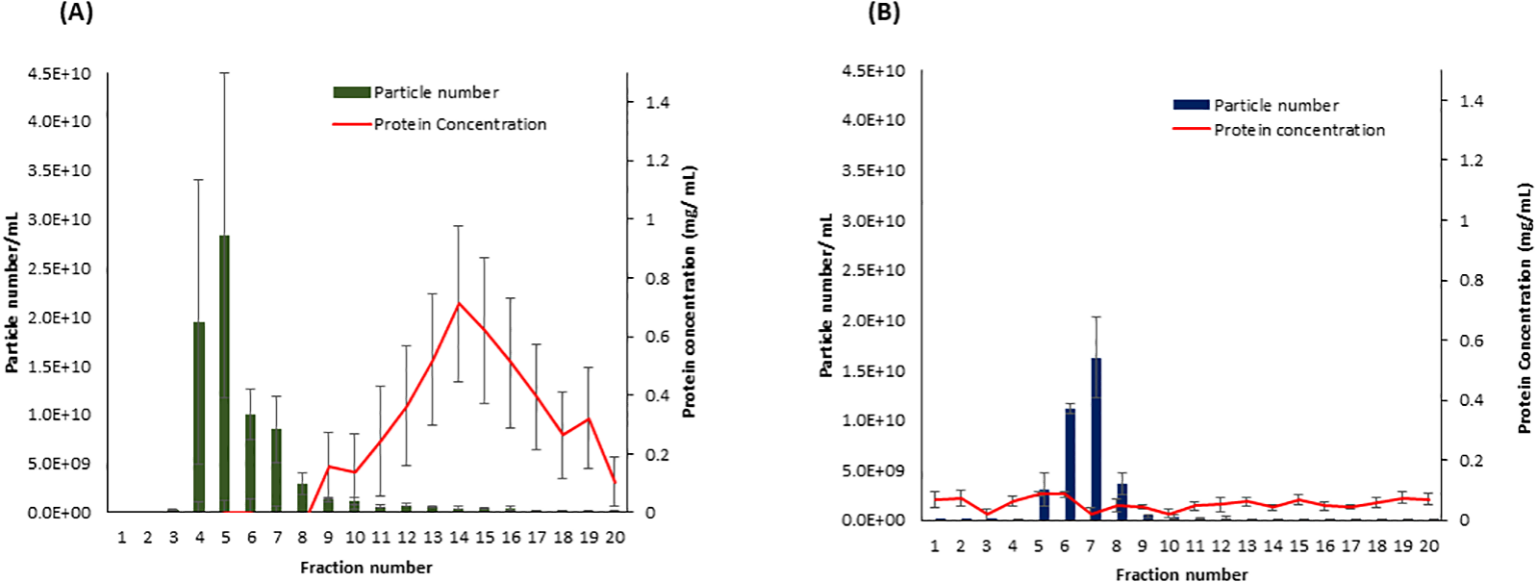
The particle and protein distribution for fractions collected from *Solanum tuberosum* (cv. Laura) peel apoplastic fluid **(A)** and root growth medium **(B)** using size exclusion chromatography (SEC). Fractions 4–8 and 5–8 contain the highest number of particles (vesicles) with a minimal amount of protein contamination for peel and root samples, respectively. n = 3; error bars represent the standard error of the mean ( ± SEM). Particle number was measured using the scatter mode of Nanoparticle Tracking Analysis (NTA), and the total protein concentration was measured using the Bradford protein assay.

To determine which SEC fractions contain the highest amount of EVs in both samples, potato peel extraction fluid (PPEF) and potato root growth medium (PRGM), 20 fractions (500 µL each fraction) were collected from each sample, and their particle concentration was measured using NTA. The NTA of potato peel and root analysis revealed a pronounced enrichment of EV fractions 4–8 and 5–8, respectively. Importantly, these fractions had minimal non-EV bound protein contamination. Notably, the protein content of PPEF sample fractions increased after the ninth fraction (**Figure 2A**), whereas PRGM samples maintained a consistent protein concentration throughout, maintaining a relatively low value of 0.10 mg/mL (**Figure 2B**). Guided by these findings, the specific fractions of 4–8 and 5–8 were collected and pooled from potato peel and root extraction fluid samples, respectively, and utilized for subsequent LC-MS/MS analysis.

### 3.2 Characterization of potato peel- and root-derived EVs

**Figure 3:**
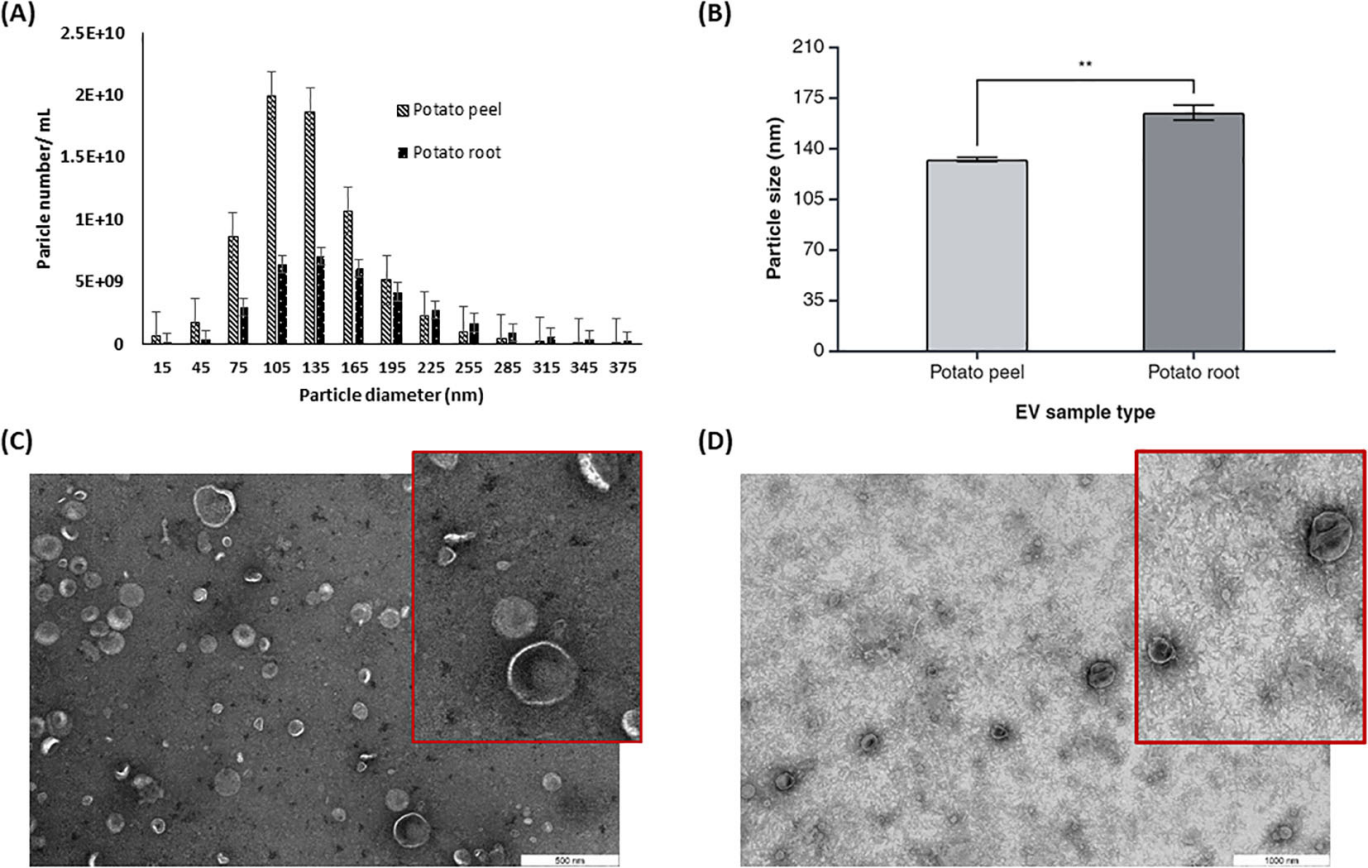
Characterization of potato peel and root derived EVs. **(A)** The particle size distribution and **(B)** mean particle size for plant derived EVs from potato peel and root measured by Zetaview® nanoparticle tracking analyzer, n=3, error bars represent the standard error of mean (± SEM, *p*= 0.0039). Asterisks (*) indicate statistical significance in particle size (nm) when comparing the potato peel and potato root sample groups, with ** denoting *p* < 0.01. **(C)** EVs purified from potato peel apoplastic fluid imaged by TEM, with the inset showing a magnified view of a selected area with a scale bar of 500 nm. **(D)** EVs purified from potato root exudates containing medium imaged by TEM, with the inset showing a magnified view of a selected area. Scale bar: 1,000 nm.

The size and morphology of potato peel- and root-derived EVs were determined by NTA and TEM. NTA results showed that the majority of these EVs’ size distribution falls in the range of 75–300 nm in diameter (**Figure 3A**). NTA data showed that the EVs released from potato root were larger than potato peel-derived EVs. The calculated values of the average cumulative size of EVs were 164.6 ± 7.3 nm and 132.2 ± 2.0 nm for potato root-derived (n = 3) and peel-derived (n = 3) EVs, respectively (**Figure 3B**). Furthermore, the one-way ANOVA with Tukey’s multiple comparisons (*p* ≤ 0.05) indicated a significant difference in the particle sizes of these two EV types (*p* = 0.0039).

TEM analysis confirmed the presence of spherical or cup-shaped morphology in both potato peel- and root-derived EV samples. There were no visible morphological differences observed between root- and peel-derived EVs with regard to their shape (**Figures 3C, D**). Comparatively, a clearer background was observed with the potato root-derived EV samples in the TEM pictures. Aside from other shape-related resemblances, the TEM data confirmed a size difference in the EVs. This supports the NTA data, which suggests that EVs originating from the root are larger than those from the peel.

### 3.3 Mass spectrometry-based proteomic analysis of potato root- and peel-derived EVs

**Figure 4:**
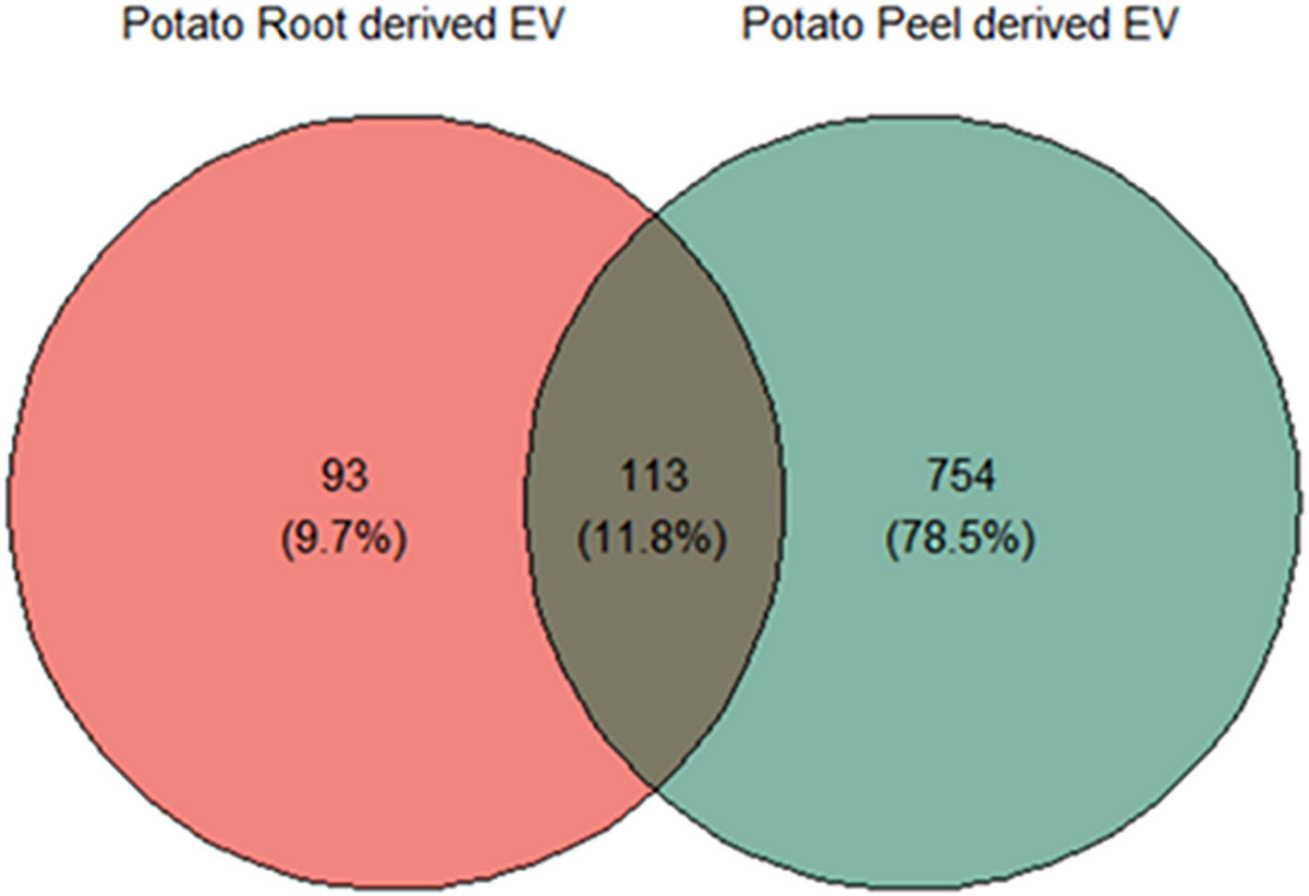
Liquid chromatography–mass spectrometry (LC-MS) and bioinformatic analysis of differentially expressed proteins; the Venn diagram shows a comparison between potato peel extraction fluid (PPEF)-derived and potato root growth medium (PRGM)-derived extracellular vesicle (EV) proteins. Out of all the proteins considered for further analysis, 93 (9.7%) were uniquely found in potato root-derived EVs, 754 (78.5%) were unique to potato peel-derived EVs, and 133 (11.8%) proteins were common to both potato peel and root samples.

Substantial variability was evident in the number of proteins identified in each crude and enriched EV sample of potato roots and peels. A total of 957 unique proteins were considered for further analysis after following the inclusion guidelines described previously. Potato root EVs were found to contain a total of 206 proteins, with 93 (9.7% of the total considered) being unique to this sample. In contrast, potato peel EVs exhibited a more diverse protein profile, with 867 proteins identified. Of these, 754 (78.5% of the total considered) were exclusive to the peel EV sample. The overlap contained 11.8% of the total proteins considered (113 proteins), which were observed to be commonly found in both sample types (**Figure 4**).

The probability of the presence of a signal peptide in proteins found in EV samples was calculated using the combined transmembrane topology and signal peptide predictor “Phobius” (Käll et al., 2004). Only 21.03% of the proteins enriched in potato peel-derived EV samples carried a signaling peptide, while 23.59% of proteins enriched in potato root-derived EV samples carried a signaling peptide. These observations reinforce the non-secretory nature of the vesicles enriched from both potato peels and roots (Regente et al., 2017; Rutter and Innes, 2017, 2020).

**Figure 5:**
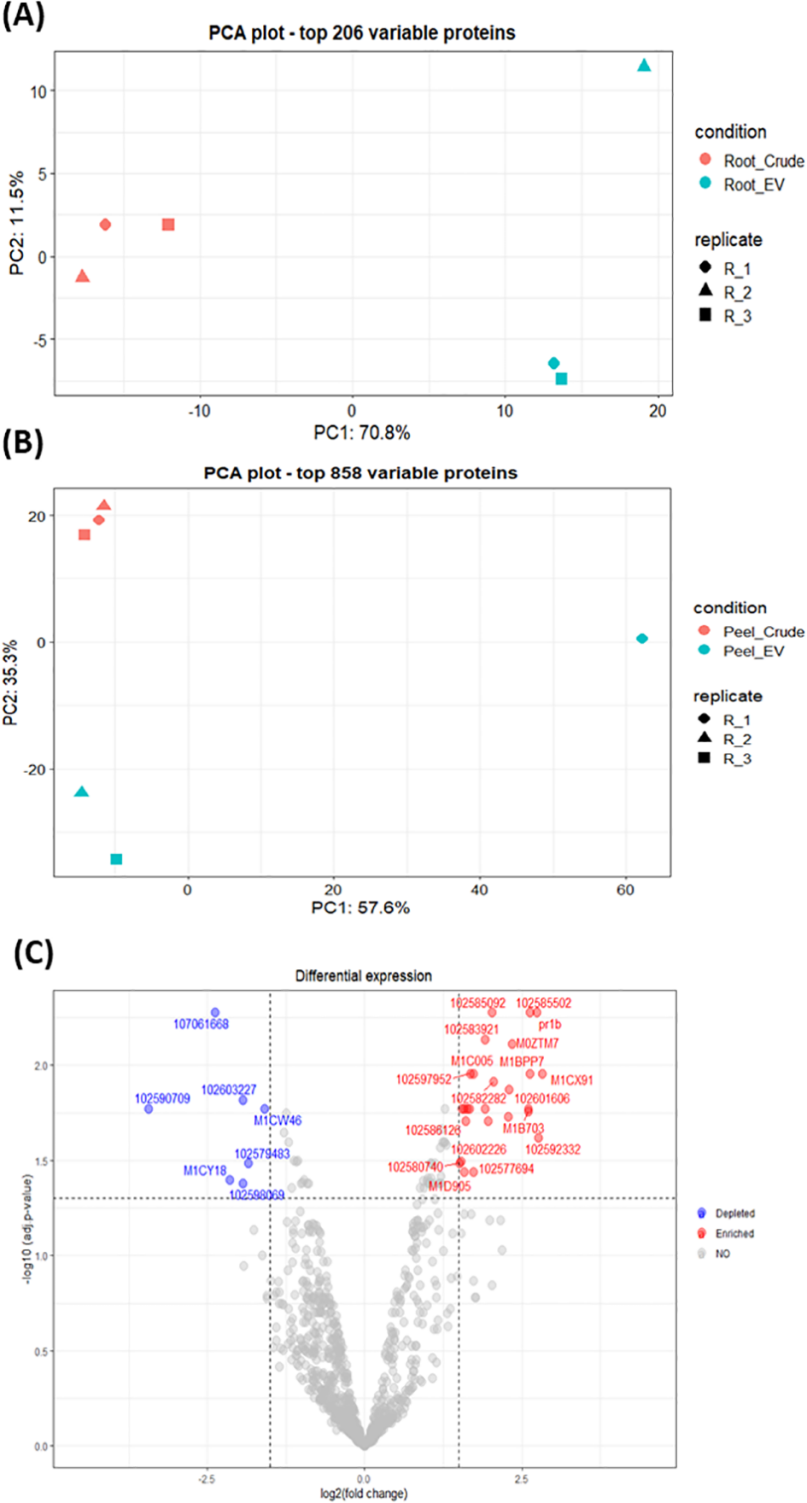
**(A)** Principal component analysis (PCA) of the 206 most variable proteins in potato root-derived extracellular vesicles (EVs) from purified (n = 3) and crude (n = 3) samples. **(B)** PCA of the 858 most variable proteins in potato peel-derived EVs from purified (n = 3) and crude (n = 3) samples. **(C)** Volcano plot visualizing differences in protein enrichment in purified potato peel EVs, with proteins enriched compared to crude samples shown in red and those depleted compared to crude samples shown in blue. Cut-off values are set at log2 fold changes of 1.5 and an adjusted *p*-value of 0.05. In total, 37 differentially enriched proteins were identified.

**Figure 6:**
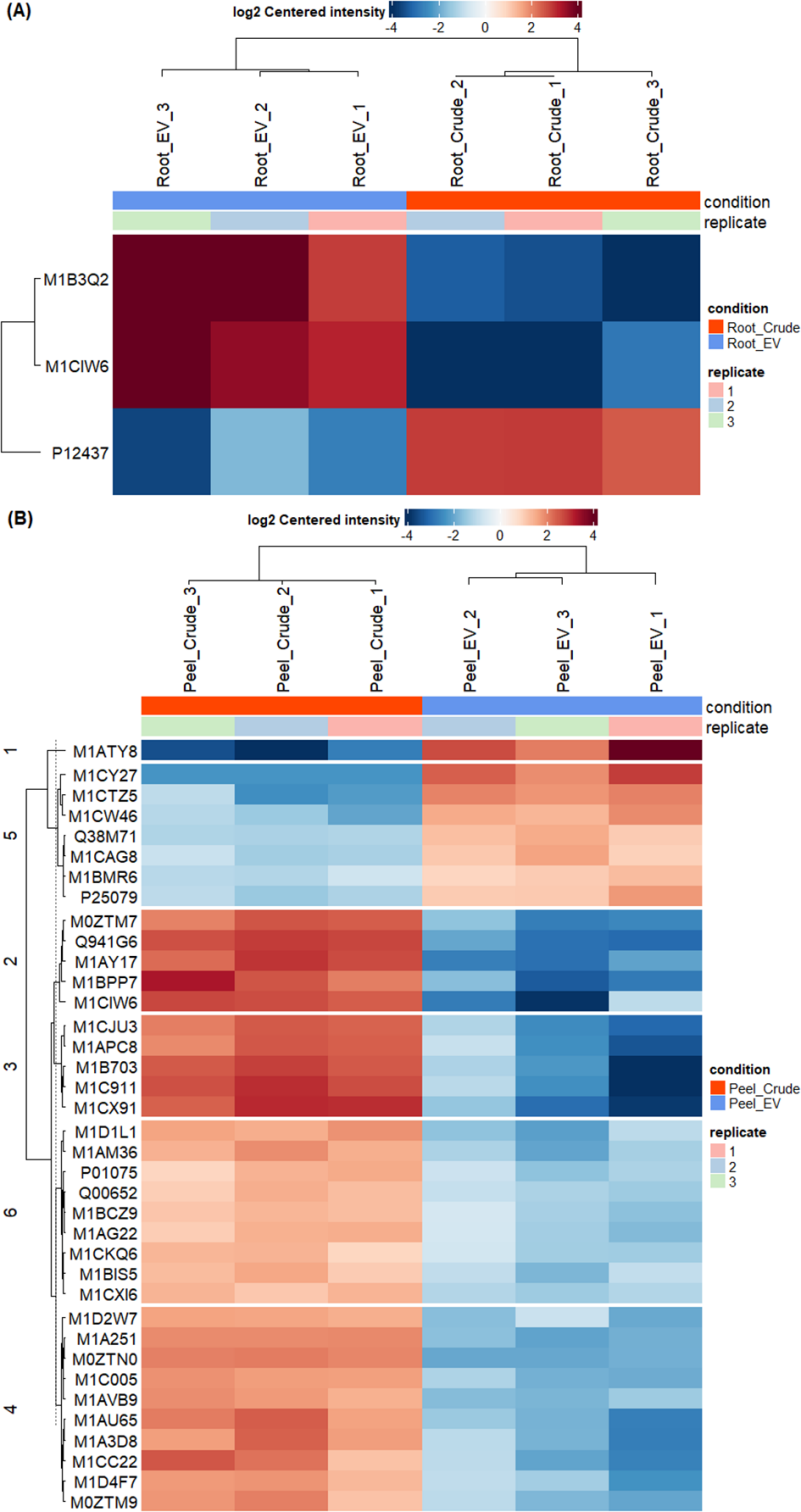
Differential enrichment analysis of extracellular vesicles from potato roots and peels. **(A)** In total, three differentially enriched proteins were identified in root-derived extracellular vesicles (EVs). The heatmap illustrates significant proteins (*p* ≤ 0.05) detected in purified (n = 3) and crude (n = 3) root-derived EV samples. **(B)** The heatmap presents significant proteins (*p* ≤ 0.05) identified in purified (n = 3) and crude (n = 3) peel-derived EV samples, showing differences in protein enrichment between the two conditions.

The differential enrichment analysis revealed distinct disparities in protein enrichment between purified EV samples to their respective crude samples in both potato root- and peel-derived EVs, respectively (**Figure 5**). The principal component analysis (PCA) further corroborated this distinction, showcasing a clear segregation between the groups of crude and purified samples for both potato peel and root EVs (**Figures 5A, B**), despite the presence of an outlier within the purified EV samples in both cases. The heatmaps drawn with significantly differentially enriched (*p* ≤ 0.05) proteins from both potato peel- and root-derived EVs vividly illustrated discernible variations in their protein enrichment patterns with respect to their condition type (crude or purified) as well as their sample type (potato root, **Figure 6A**, or peel, **Figure 6B**). Following enrichment, a total of 37 proteins were recorded in potato peel EV samples, whereas root EV samples exhibited three differentially enriched proteins (*p* ≤ 0.05).

There was minimal overlap in differentially enriched proteins between potato root and peel. Only two proteins (2%) out of the enriched proteins were identified as common between the two groups. A single protein was detected to be depleted in both potato root- and peel-derived EVs compared to their respective crude samples, while 49 proteins remained unchanged in both EV samples (**Supplementary Figure 1**). Notably, thioredoxin-dependent peroxiredoxin (M1CQK1) and adenylosuccinate lyase (M1CMF9) emerged as consistently enriched proteins in both root and peel samples.

**Figure 7:**
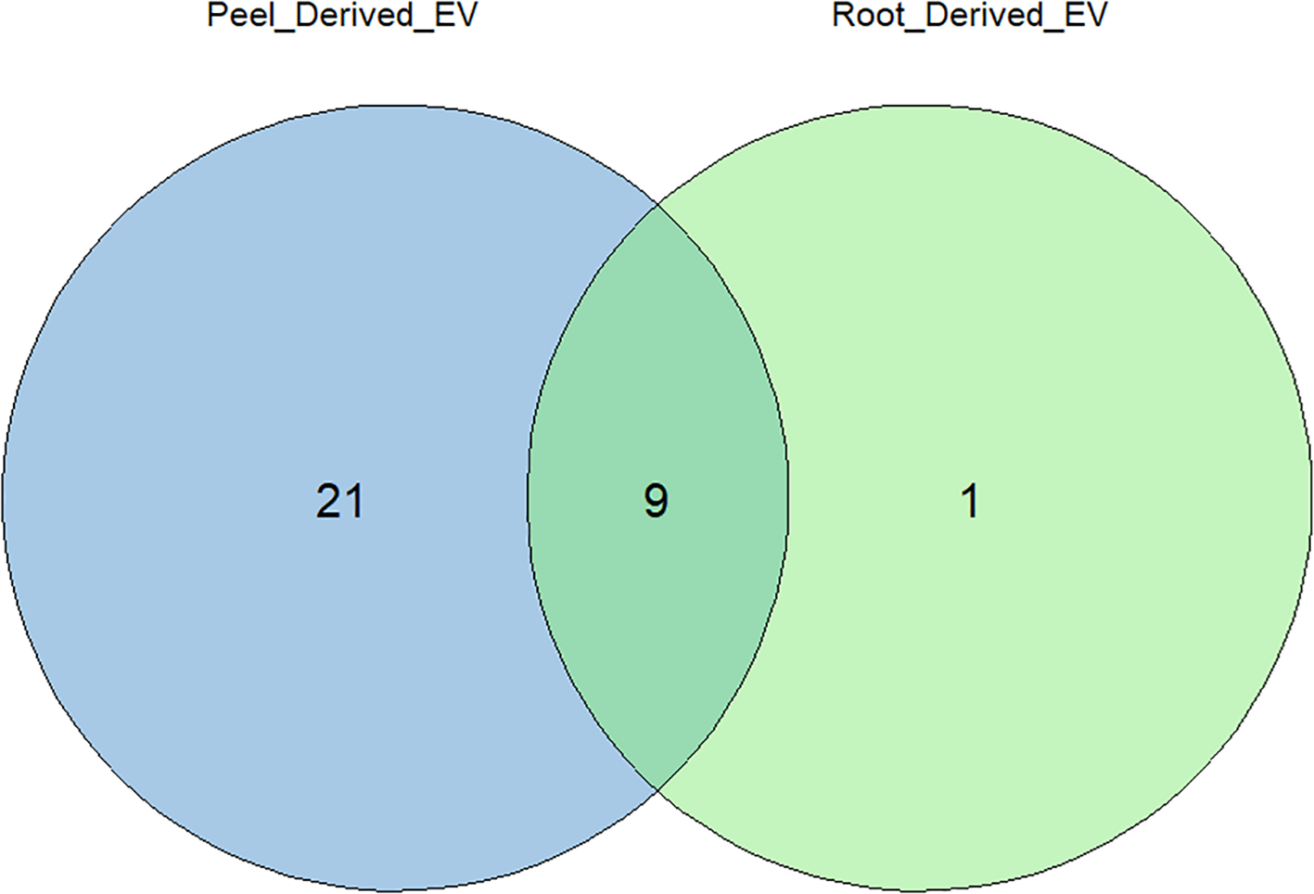
Distribution of identified extracellular vesicle (EV) protein markers between the proteomes of potato peel- and potato root-derived EVs. Out of the 30 EV markers considered, 19 were uniquely found in potato peel-derived EVs, and one EV marker was uniquely found in potato root-derived EVs. The remaining nine markers were equally present in both samples.

From the comparative analysis of the enriched proteome obtained in this study with the most recent recorded work, several potential plant EV protein markers were identified. Among these, the most commonly observed proteins included TET8, PEN3, HSP70, HSP90, and PATL1, which are known markers of plant EVs (**Table 1**). These 30 proteins were consistently recorded across studies, indicating their potential as reliable markers for plant-derived extracellular vesicles. This alignment with the existing literature strengthens the relevance of our findings and further suggests that these proteins play key roles in EV-mediated processes. **Figure 7** describes the distribution of the identified protein markers among the potato peel and potato root samples.

**Table 1:** The presence of the 30 most reported EV protein markers found in potato peel- and root-derived EVs.

	UniProt protein ID (*Solanum tuberosum*)	Protein name	Presence in potato peel EVs	Presence in potato root EVs	References
1	M1AM36	Pectinesterase	+	+	(Woith et al., 2021; Rutter and Innes, 2017)
2	M0ZMG2	Class II chitinase	+	+	(Regente et al., 2017; De Palma et al., 2020)
3	M1CS26	Phospholipase D (EC 3.1.4.4)	+	−	(Rutter and Innes, 2017)
4	M1BBH3	Patellin-3	+	−	(Regente et al., 2017; Rutter and Innes, 2017; Pocsfalvi et al., 2018)
5	M1AKD9	Heat shock protein 83	+	−	(Saito et al., 2024)
6	M0ZXL7	Actin	+	−	(Woith et al., 2019, 2021; Boccia et al., 2022)
7	M1BQI2	Heat shock protein 90	+	−	(Rutter and Innes, 2017)
8	P30171	Actin-97 (EC 3.6.4.-)	−	+	(Woith and Melzig, 2019)
9	M1ARI6	Phosphopyruvate hydratase (Enolase) (EC 4.2.1.11)	+	−	(Timms et al., 2019; Boccia et al., 2022)
10	M1AS40	Senescence-associated protein—TET8	+	+	(Cai et al., 2018; He et al., 2021; Chaya et al., 2024)
11	M1AHN5	Heat shock protein 70	+	−	(Movahed et al., 2019; Yugay et al., 2023)
12	M1AWY0	Aquaporin PIP-type pTOM75	+	−	(De Palma et al., 2020)
13	M1AUJ0	Alpha-galactosidase (EC 3.2.1.22) (Melibiase)	+	−	(Woith et al., 2021)
14	M1AGK5	Endochitinase (Chitinase)	+	−	(De Palma et al., 2020; Woith et al., 2021)
15	M1CC22	Peroxidase (EC 1.11.1.7)	+	+	(Regente et al., 2017)
16	P52404	Endochitinase 2 (EC 3.2.1.14)	+	+	(De Palma et al., 2020)
17	M1CJU3	Endochitinase 4	+	+	(De Palma et al., 2020)
18	M1A2M6	Patellin 1	+	−	(Regente et al., 2009; Rutter and Innes, 2017; Pocsfalvi et al., 2018; Chaya et al., 2024)
19	M1AGX9	Tubulin alpha chain	+	−	(Boccia et al., 2022)
20	M1ARQ6	Tubulin beta chain	+	+	(Boccia et al., 2022)
21	M1AUY9	Chitinase	+	+	(De Palma et al., 2020)
22	M1BV08	Aquaporin	+	−	(Oshikawa et al., 2016; De Palma et al., 2020)
23	M0ZRE9	Phosphotransferase (EC 2.7.1.-)	+	−	Rutter, Brian D. Indiana University ProQuest Dissertations & Theses, 2019. 13808326.
24	M1AW19	Polygalacturonase	+	−	(Yugay et al., 2023)
25	M0ZNV9	Annexin	+	−	(Rutter and Innes, 2017)
26	M0ZRW4	PDR8/PEN3 (Pleiotropic Drug Resistance8)	+	−	(Rutter and Innes, 2017; Chaya et al., 2024)
27	M1CE12	Profilin	+	−	(Timms et al., 2019)
28	M1BEU2	Serine-threonine protein kinase, plant-type	+	+	(De Palma et al., 2020)
29	M1C5H3	Epoxide hydrolase	+	−	(Woith et al., 2021)
30	M1D0R2	Glycosyl hydrolase family 3 protein	+	−	(Woith et al., 2021)

EVs, extracellular vesicles.

The symbols “+” and “−” indicate the presence and absence, respectively, of the given protein markers in potato peel and root-derived EVs.

### 3.4 Functional bioinformatic analysis of EV proteomes of potato peels and roots

The gene set enrichment analysis (GSEA) identified a total of 342 enriched proteins in potato peel-derived EVs and 108 enriched proteins in root-derived EVs. The most highly enriched proteins for each sample are presented in **Table 2** (peel-derived EVs) and **Table 3** (root-derived EVs), showcasing key proteins involved in various biological processes. The remaining enriched proteins from both the peel and root EVs are provided in the **Supplementary Material** for further reference.

**Table 2:** Highly enriched functional proteins in peel-derived EVs identified through differential protein enrichment analysis.

	ID	Description	Log fold change
1	M1BP14	60S ribosomal protein L8	3.7
2	M1CW46	Polyphenol oxidase	3.17
3	M1BCL1	26S proteasome regulatory particle non-ATPase subunit 8	2.74
4	M1CK73	Pyruvate dehydrogenase	2.65
5	M1BCJ2	Prefoldin subunit	2.59
6	Q38M71	UDP-glucose:protein transglucosylase	2.5
7	M1A0X0	Multicopper oxidase	2.44
8	M1CAG8	GRP 2	2.42
9	M0ZWN3	Cysteine protease	2.32
10	M1BQ74	Ribulose bisphosphate carboxylase small chain 1, chloroplastic	2.3
11	M1D253	Nucleoredoxin	2.3
12	K7XKT7	Alpha-1,4-glucan-protein synthase [UDP-forming] 1	2.2
13	M1CQK3	Thioredoxin peroxidase	2.2
14	M1AQZ1	Pyruvate kinase, cytosolic isozyme	2.11
15	M1BMR6	Polyphenol oxidase	2.11
16	M1AE81	Major intrinsic protein 2	2.09
17	M1CF76	Cinnamyl alcohol dehydrogenase	2.06
18	M1AQ24	Methylmalonate-semialdehyde dehydrogenase	2.05
19	M1C3W1	Inactive purple acid phosphatase 27	2.03
20	M1C9T0	Aldehyde dehydrogenase	2.01
21	M1D7J7	Malic enzyme	2.01
22	M1AHN5	Heat shock protein 70	1.98
23	M1B818	Aspartyl aminopeptidase	1.98
24	M1CEF5	Ribosomal protein	1.98
25	M1CY58	Ascorbate peroxidase	1.89

The table presents 25 selected proteins deemed to be functionally important using GSEA, showing their description and enrichment ratio (log fold change), which represents the enrichment of EV proteins compared to crude potato peel samples. A full set of proteins used for differential enrichment analysis can be found in **Supplementary Table 1**. Proteins that were found to be functionally important using GSEA are listed in **Supplementary Table 3**.

EVs, extracellular vesicles; GSEA, gene set enrichment analysis.

**Table 3:** Highly enriched functional proteins in root-derived EVs identified through differential protein enrichment analysis.

	ID	Description	Log fold change
1	M1CIW6	Class III peroxidase	7.59
2	M1B3Q2	Peroxidase	7.54
3	M1AQK9	Conserved gene of unknown function	4.56
4	M1A2A5	Basic PR-1 protein	4.45
5	M1AG22	Miraculin	4.19
6	M0ZMG2	Class II chitinase	3.79
7	M1AN26	Miraculin	3.67
8	M1AW41	Dirigent protein	3.63
9	M1A2Z2	Peroxidase	3.53
10	M1AE23	Protein Z	3.39
11	M1BLS0	Multicopper oxidase	3.33
12	Q941G6	Cytoplasmic small heat shock protein class I	3.23
13	M1AGX9	Alpha-tubulin	3.06
14	M1AQ00	Superoxide dismutase [Cu-Zn] 2	3.02
15	M1CB98	Serine carboxypeptidase	2.96
16	M1AUY9	Chitinase	2.68
17	M1CLQ7	Serine carboxypeptidase-like 27	2.6
18	M0ZI69	Isocitrate dehydrogenase [NADP]	2.58
19	M1A2A2	PR-1	2.56
20	M0ZPP3	Pectinesterase	2.55
21	M1CQK3	Thioredoxin peroxidase	2.47
22	M1AGX5	Patatin-05	2.41
23	M1D1U1	Ribosomal protein	1.98
24	M1BN40	Ribosomal protein PETRP	1.91
25	M1AMY2	Ascorbate peroxidase	1.89

The table presents 25 selected proteins deemed to be functionally important using GSEA, showing their description and enrichment ratio (log fold change), which represents the enrichment of EV proteins compared to crude potato root samples. A full set of proteins used for differential enrichment analysis can be found in **Supplementary Table 2**. Proteins that were found to be functionally important using GSEA are listed in **Supplementary Table 4**.

EVs, extracellular vesicles; GSEA, gene set enrichment analysis.

**Figure 8:**
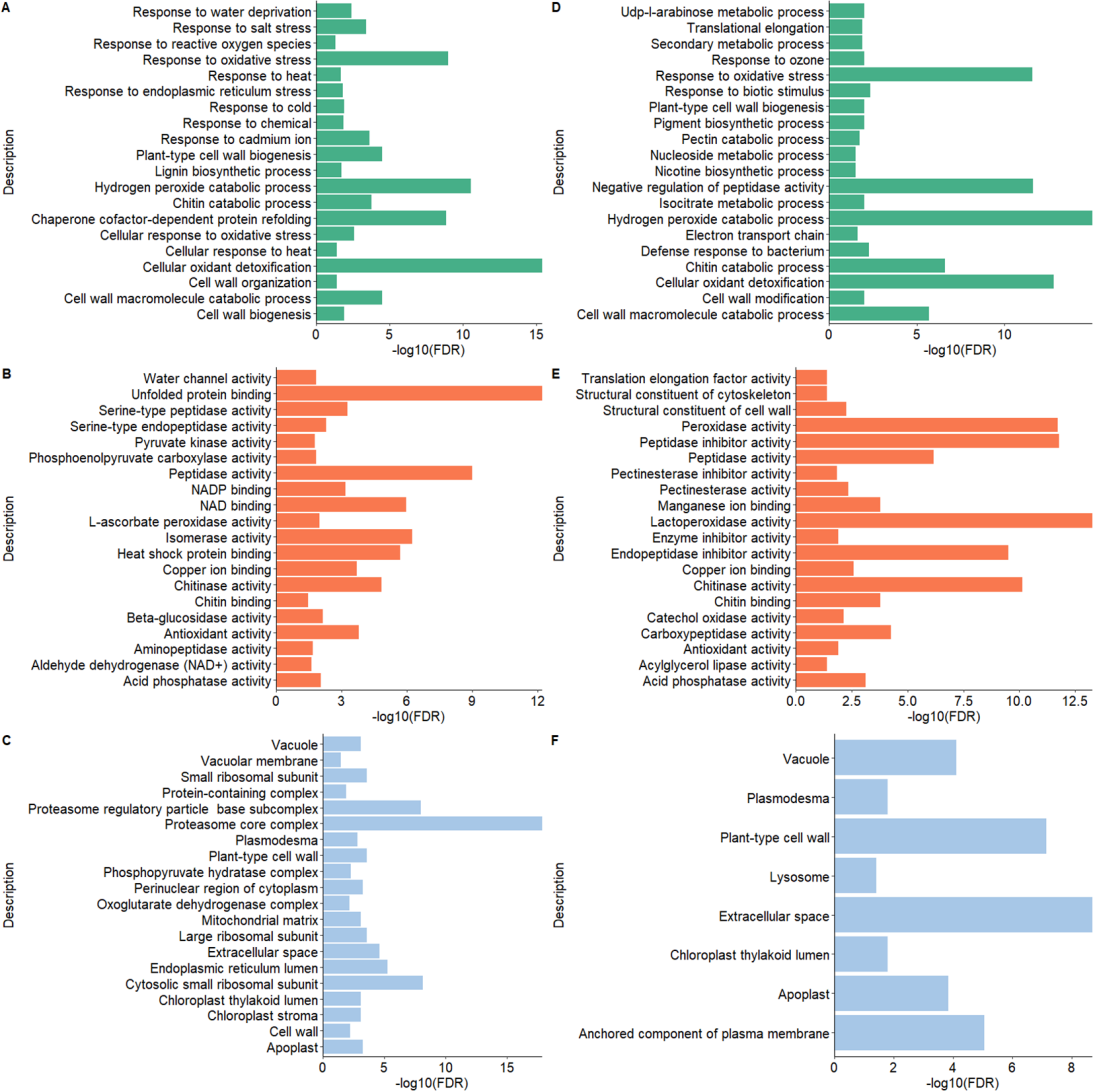
Gene Ontology (GO) functional enrichment analysis of the proteomes of extracellular vesicles (EVs) derived from potato peel **(A–C)** and potato root **(D–F)**. **(A, D)** show the biological processes, panels **(B, E)** depict the molecular functions, and **(C, F)** illustrate the cellular components. The GO terms for each EV sample were ranked according to their enrichment score [-log_10_(FDR)], highlighting the most relevant GO terms within each dataset.

The pathway enrichment analysis employing GO terms unveiled notable findings about cellular components, molecular functions, and biological processes for both potato peel- and root-derived EV proteomes. In terms of biological processes (BPs) under GO terms, the EV proteins shared between potato peels and roots displayed a significant association with response to various stress factors, including water, temperature, salt, chemicals, and oxidative stress. The significance of these associations was determined by *p*-values calculated from FDR values. Stress factors were considered statistically significant when the *p* < 0.005 in correspondence to the −log10 (FDR) value. In addition, these proteins were found to be localized in cellular components such as cell walls, cytoplasm, plasmodesma, and apoplast of plant cells. Furthermore, several molecular functions (MFs) were identified, encompassing activities such as chitinase activity and antioxidant activity, as well as structural roles as constituents of the cell wall and cytoskeleton (**Figure 8**).

## 4 Discussion

The characterization data demonstrate the success of the EV enrichment process used in the current study. Despite the challenges in collecting samples, particles closely resembling EVs in terms of shape, size, composition, and quantity were successfully enriched and characterized using multiple characterization techniques such as NTA, TEM, and proteomics. The observed size range of the enriched particles aligns with the typically attributed size range of plant-derived EVs, as defined by the latest guidelines proposed by the ISEV (Pocsfalvi et al., 2018; Lian et al., 2022; Welsh et al., 2024). The consistency of the results obtained through these comprehensive analyses confirms the successful enrichment of EVs, reinforcing the reliability and relevance of the study findings.

The distinct size profile differences observed between the potato root and peel EVs can be attributed to a range of factors. Among these factors, a plausible explanation for the variation in size profiles arises from the differences in cellular composition and functions inherent to root and peel tissues. Plant cells exhibit distinctive traits based on the tissue type, and these variations exert an influence on the size and composition of EVs released from different plant parts (Parre and Geitmann, 2005; Nemati et al., 2022). Root tissues are primarily involved in nutrient absorption and anchoring the plant, while peel tissues fulfill a protective role. These distinct functionalities could result in variations in the content and packaging of EVs released by these tissues. Furthermore, the differences in the mechanical properties between root and peel tissues could also contribute to the observed variation in EV size profiles. Physical properties of cells, such as cell wall rigidity and elasticity, can affect the release and size distribution of EVs (Ambrosone et al., 2023). Root tissues, being responsible for water and nutrient uptake, may possess dissimilar mechanical characteristics compared to protective peel tissues. These discrepancies in tissue mechanics may influence the release and subsequent size distribution of EVs. Additionally, the specific developmental stages of the root and peel tissues at the time of EV isolation could impact the size profiles. Plant tissues undergo dynamic changes during development, including alterations in cell size, division, and expansion. Thus, these developmental processes have the potential to influence the release and composition of EVs (Kondhare et al., 2020). Therefore, differences in the developmental stages between root and peel tissues may contribute to the observed variations in EV size profiles.

The crude and enriched EV samples exhibited distinct proteomic profiles, indicating successful enrichment of the EVs from crude preparations. The term “EVs” was used for the particles found to be enriched given the high resemblance that they had to mammalian EVs. This observation aligns with the findings of other researchers and supports the inclusion of plant-derived particles as EVs, as previously suggested by Lian et al. (2022). The field of plant EVs lacks the organizational structure present in mammalian EV research, particularly in terms of concrete guidelines for confirming particles as EVs. Even international bodies like the ISEV have not proposed definitive guidelines for characterizing plant EVs, especially concerning protein markers. The lack of universal markers for plant EVs is notable. Nonetheless, the enriched EV samples have revealed the presence of proteins such as enolase (phosphopyruvate hydratase), heat shock proteins (HSP70 and HSP90), patellin proteins (PATL1 and PATL3), tetraspanin (TET8), penetration protein (PEN3), and chitinases including endochitinases. These proteins have been identified in previous studies as prevalent in plant EVs (Regente et al., 2017; Movahed et al., 2019; Timms et al., 2019; Woith and Melzig, 2019; De Palma et al., 2020; Yugay et al., 2023). These proteins emerge as potential candidates for consideration as universal markers for plant EVs.

Interestingly, proteins such as TET8, chitinases, and endochitinases were detected in both peel- and root-derived enriched EVs. However, other proteins, including PEN3, PATL1, and PATL3, were exclusively found in the potato peel samples. Additionally, Actin-97 was only identified in the potato root samples.

The current study further revealed minimal overlap in enriched proteins between potato root and peel samples, with only two proteins (2%) identified as common to both groups. These shared proteins were thioredoxin-dependent peroxiredoxin (M1CQK1) and adenylosuccinate lyase (M1CMF9). These proteins primarily contribute to plant stress responses and catalytic activities. Previous studies have recorded that thioredoxin-dependent peroxiredoxin is important for cellular antioxidant defense, showing consistent enrichment, and indicating active shielding against oxidative stress, which is vital for plant survival. Conversely, adenylosuccinate lyase participates in purine metabolism, facilitating the conversion of succinyl adenosine monophosphate (SAMP) to AMP and fumarate. The enrichment in both root and peel samples highlights the significant contribution of these proteins in mitigating oxidative stress and facilitating enzymatic reactions critical for plant survival and adaptation to environmental challenges (Siehl et al., 1996; Zhang et al., 2020).

A relatively low percentage of proteins enriched in the EV samples in the current investigation carried signal peptides (21.03% for peel-derived EVs and 23.59% for root-derived EVs). Multiple previous studies have hypothesized that the low percentage of proteins that carry signal peptides is evidence of the “non-secretory” nature of plant EV. This phenomenon could also be considered evidence for the lack of contamination by cellular debris and secretory vesicles in the EV samples used in the current study (Regente et al., 2017; Rutter and Innes, 2017).

A number of proteins found in the present study were involved in basic metabolic processes, vesicle trafficking, and proteolysis, and represent cytoskeletal components. These proteins were also frequently present in mammalian EVs. This overlap in protein composition may reflect a conserved function and/or origin of EVs (Choi et al., 2015). Functional analysis of the enriched EVs revealed three main areas of enrichment relevant to potato plant physiology. One of the specific groups of GO terms (Biological Process) suggests that plant EVs contained proteins linked to cell wall modification or remodeling in both potato root and peel cells. This concept of cell wall modification has been proposed in multiple studies exploring the plant EV proteome (Prado et al., 2014; Regente et al., 2017; Rutter and Innes, 2017; de la Canal and Pinedo, 2018; Timms et al., 2019). Unlike mammalian cells, plant cells have cell walls that provide a barrier to EV release, either via multivesicular body fusion or plasma membrane budding. Pectin, a key component of the plant cell wall, regulates the size of the pores within the primary cell wall, which is essential for controlling the movement of molecules and particles including EVs. It has been hypothesized that the EV proteome may contribute to modifying the structure of the cell wall, potentially altering pectin crosslinking and reducing its integrity, increasing pore size, and enabling EVs to navigate into the apoplastic space (Pocsfalvi et al., 2019; Ambrosone et al., 2023). EVs may also use cell wall-modifying capabilities to enter target cells that are protected by cell walls (Rutter and Innes, 2017; Ruf et al., 2022). Proteins involved in cell wall modification were identified in both peel- and root-derived EVs, further supporting the hypothesis of cell wall reorganization. Notably, enzymes such as pectinesterase, polygalacturonase, and glycosyl hydrolase family 3 proteins were detected. These proteins are well-known for their roles in the degradation and remodeling of polysaccharides in the plant cell wall, such as pectin and cellulose (Hadfield and Bennett, 1998; Parre and Geitmann, 2005; Minic and Jouanin, 2006). Additionally, cytoskeletal proteins such as actin and tubulin were detected, which are likely to be involved in intracellular trafficking. In this context, they may facilitate the transport and delivery of vesicles, including EVs, as well as contribute to processes like cell wall deposition (Wasteneys, 2002; Szymanski and Staiger, 2018). The presence of given proteins suggests that the hypothesis of EV ability in cell wall modification and reorganization holds validity. Comparisons can be drawn between the enrichment of markers involved in EV biogenesis for both plant cell wall reorganization and the endosomal sorting complex required for transport (ESCRT) mechanism markers enriched in mammalian EVs. In line with our argument, another hypothesis worth mentioning pertains to the release of EVs by fungi and gram-positive bacteria (Lee et al., 2009; Oliveira et al., 2010). Similar to plants, these organisms also possess a thick cell wall. Notably, proteins linked to polysaccharide metabolism have been found in abundance in EVs isolated from these organisms. This finding implies that the process of the EVs crossing the cell wall may necessitate cell wall remodeling through an unknown mechanism (Brown et al., 2015).

Another set of GO terms related to molecular functions that were enriched in both potato peel- and root-derived EVs revolved around biotic and abiotic stress factors. In plants, biotic stress primarily addresses cellular defense mechanisms against pathogens such as bacteria, viruses, and fungi, while abiotic stress factors encompass conditions like water stress, cold and heat, oxidative stress, and exposure to toxic chemicals. The presence of proteins such as endogenous chitinases (class II chitinases and endochitinases) and basic pathogenesis-related (PR) proteins is crucial for biotic stress responses, particularly in defense against fungal pathogens (Santos et al., 2017; Kumar et al., 2018). Additionally, the presence of peroxidases like ascorbate peroxidase, thioredoxin peroxidase, multicopper oxidase, and HSP70, which help mitigate oxidative stress and maintain cellular homeostasis during abiotic stress, further supports this role (Passardi et al., 2005; Berka et al., 2022; Chen et al., 2023). Plant-derived EVs have been implicated in cellular defense ever since their discovery in plants (Halperin and Jensen, 1967). They are considered to be integral components of a plant’s cellular defense system. Growing evidence demonstrates that many plant EVs contribute to plant immunity by mediating the transport of regulatory small RNAs into pathogens, resulting in the suppression of genes associated with pathogen virulence (Regente et al., 2017; Cai et al., 2018, 2021; Cai et al., 2019; Liu et al., 2021). It is noteworthy that prior findings by Rutter and Innes (2020) support the notion that plant EV production is enhanced in response to both biotic and abiotic stress, with EVs being enriched in defense- and stress-related proteins (Rutter and Innes, 2020). Their study employed the whole EV proteome of the model plant *Arabidopsis thaliana*. While the present study and its proteomic analyses were conducted under stress-free growth conditions, the protein composition of both potato peel and root EVs strongly indicates their involvement in plant innate immunity. This finding aligns with the studies conducted by Regente et al. (2017) on *Helianthus annuus* L. and Rutter and Innes (2017) on *A. thaliana*, both of which similarly suggested the involvement of EVs in plant innate immunity (Regente et al., 2017; Rutter and Innes, 2017). Furthermore, a study by De Palma et al. (2020) demonstrated the antifungal activity of EVs derived from tomato roots, showing significant inhibition of spore germination and germination tube development in various fungal pathogens, including *Fusarium oxysporum*, *Botrytis cinerea*, and *Alternaria alternata* (De Palma et al., 2020). These findings further prove that EVs contribute to innate immunity through active defense mechanisms. Incorporating this, we can argue that the enrichment of defense-related proteins in EVs, even under non-stressful conditions, strengthens the hypothesis that EVs are fundamental components of innate immunity across different plant species. This aspect of plant-derived EVs is particularly intriguing due to its potential for practical applications in the field of agriculture. Plant-derived EVs could be harnessed as therapeutic agents against plant pathogens and other stressors that may affect plant cells.

Chloroplastic proteins are the other subpopulations that appeared to be well-represented in potato peel- and root-derived EVs. A considerable proportion of these proteins may be responsible for cell leakage or participate in remodeling processes during stress response, contributing to the shaping of the plant’s peptidome (Mamaeva et al., 2020). Interestingly, there is a compelling hypothesis suggesting that EVs could play a role in cellular-level waste management (Regente et al., 2017). Autophagy has been established as a cellular mechanism for chloroplast degradation (Xie et al., 2015), and EVs may contribute to the removal of waste products linked with autophagy (Baixauli et al., 2014; Xu et al., 2018). The substantial presence of proteases detected in both potato peel- and root-derived EVs in this study may also be related to significant degradation and recycling mechanisms in plants that contribute to the maintenance of cellular homeostasis—a process that is not yet fully understood. While it remains speculative to predict the precise function of these EVs based solely on the protein data collected, these findings may offer valuable insights for future investigations.

A comprehensive characterization study of EVs like this one from potato root and peel tissues offers valuable insights into the mechanisms underlying plant physiology and defense mechanisms, which can significantly impact agricultural practices and crop productivity. First, the successful enrichment and characterization of EVs provide a reliable foundation for further research into the role of EVs in plant–microbe interactions, nutrient uptake, and stress responses. Second, the observed differences in EV size profiles between root and peel tissues highlight the importance of tissue-specific differences in cellular composition and functions. These observations describe the diverse roles of EVs in plant development and responses to environmental stimuli and suggest that, with further research, strategies could be developed to optimize crop growth and resilience to stressors by exploring tissue-specific EV pathways, which remain to be fully understood.

The identification of distinct proteomic profiles in crude and enriched EV samples shows the potential for using EVs as biomarkers of crop health and stress tolerance. The presence of conserved proteins involved in basic metabolic processes, vesicle trafficking, and cytoskeletal components suggests evolutionary similarities in the origin and function of EVs across plant and mammalian systems, which could guide the development of novel biotechnological applications and solutions in various sectors, including agriculture and health.

The present study acknowledges certain aspects that warrant attention. First, the methods employed for extracting EVs from potato peel and root samples entail limitations. During SEC, some non-EV-related particles were inevitably co-isolated with the EVs, posing a challenge in effectively separating non-EV particles from EVs of the same size. To the best of our knowledge, there is currently no existing isolation method that can distinguish between them. Second, it is important to note that plant-derived EVs typically contain a significantly lower quantity of proteins compared to mammalian EVs. As a result, obtaining a sufficiently ample amount of protein for comprehensive and robust proteomic analyses becomes increasingly challenging. Third, there is a drastic difference in the number of proteins between the potato root-derived EV samples and the potato peel-derived EV samples. These differences could be attributed to the nature of the sample, with the crude sample for potato peel (apoplastic fluid) being a highly concentrated source of particles compared to the vastly more diluted hydroponic media that was the crude sample for the potato root-derived EVs. Longer concentration steps were required to process the potato root-derived EVs, leading to higher particle and protein losses due to degradation and handling losses. Finally, it is crucial to acknowledge that the plant proteomes, including their associated gene ontology terms, generally exhibit a lesser degree of definition and detail compared to mammalian proteomes and genomes. This disparity increases the likelihood of potentially overlooking significant connections between observed proteomic enrichments.

Taken together, these findings demonstrate the feasibility of enriching EVs from root cells and periderm/peel of potato tubers, thus presenting a potential and sustainable source of EVs. Although visually similar, EVs from the two sources are significantly different in size profile and protein content. A proteomic analysis revealed that EVs released by potato roots and peels carry proteins associated with plant cell remodeling and responses to biotic and abiotic stress factors, indicating their potential involvement in plant defense mechanisms. These observations reveal that with further development, potato root- and peel-derived EVs may have the potential to be used as agents in plant defense. However, further investigations are required to unravel their mechanisms within plant cells and EVs, as well as their potential roles in inter-kingdom communication. Furthermore, in the domain of plant waste valorization, the standardized characterization of potato root- and peel-derived EVs is the initial step in developing a mass-produced biomaterial with potential industrial uses.

## Data Availability

The datasets presented in this study can be found in online repositories. The names of the repository/repositories and accession number(s) can be found below: https://figshare.com/, https://doi.org/10.6084/m9.figshare.25607601.v1.
