# Supplementary figures and images for: Systematic characterization of extracellular vesicles from potato (Solanum tuberosum cv. Laura) roots and peels: biophysical properties and proteomic profiling
Source: Front Plant Sci. 2024 Nov 15;15:1477614. doi: 10.3389/fpls.2024.1477614 (PMC11607679; doi:10.3389/fpls.2024.1477614)

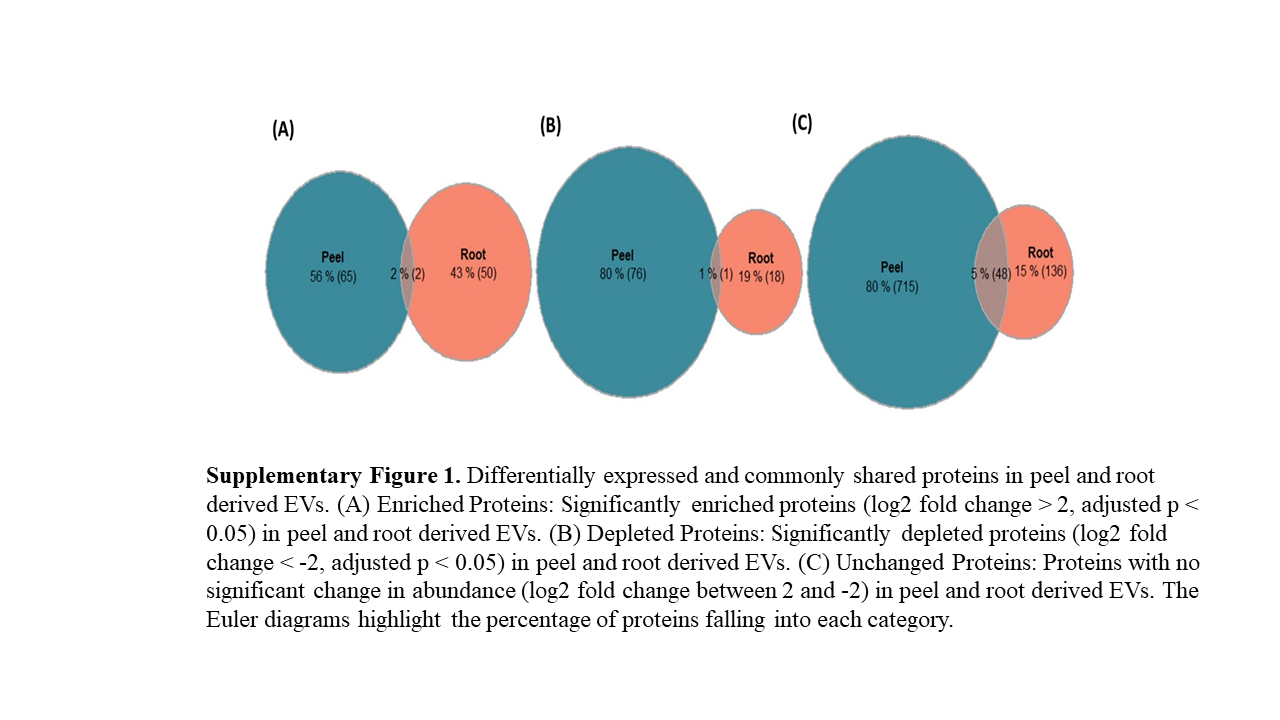

Supplement: Supplementary file 5 [file Image1.tif]
