# Supplementary material for: Systematic characterization of extracellular vesicles from potato (Solanum tuberosum cv. Laura) roots and peels: biophysical properties and proteomic profiling
Source: Front Plant Sci. 2024 Nov 15;15:1477614. doi: 10.3389/fpls.2024.1477614 (PMC11607679; doi:10.3389/fpls.2024.1477614)
Supplement: Supplementary file 6 [file Table1.docx]

Supplementary Material

# Supplementary Figures and Tables legends

## Supplementary Tables

**Supplementary Table 1. Differential enrichment analysis of peel derived EV proteins compared to crude**

A total of 858 unique proteins were used for the differential enrichment analysis using the R package Differential Enrichment analysis of Proteomics data (DEP). The log2 transformed LFQ intensity values were used for proteins detected in at least two out of three replicates of either crude or EV protein samples. In the analysis, the k-nearest neighbour approach (knn) was used for data imputation. Relative enrichment (log_2_ fold change) of the peel EV proteins compared to the crude sample (Purified_EV_vs_Crude_ratio) and the adjusted p value (Purified_EV_vs_Crude_p.adj) are presented in the table along with the details of proteins. Protein enrichments were considered significant when adjusted *p* value (Benjamini-Hochberg method) < 0.05.

**Supplementary Table 2. Differential enrichment analysis of root derived EV proteins compared to crude**

A total of 206 unique proteins were used for the differential enrichment analysis using the R package Differential Enrichment analysis of Proteomics data (DEP). The log2 transformed LFQ intensity values were used for proteins detected in at least two out of three replicates of either crude or EV protein samples. In the analysis, the k-nearest neighbour approach (knn) was used for data imputation. Relative enrichment (log_2_ fold change) of the peel EV proteins compared to the crude sample (Purified_EV_vs_Crude_ratio) and the adjusted p value (Purified_EV_vs_Crude_p.adj) are presented in the table along with the details of proteins. Protein enrichments were considered significant when adjusted *p* value (Benjamini-Hochberg method) < 0.05.

**Supplementary Table 3. Potato peel proteins enriched in network analysis**

Network analysis was performed on differentially expressed proteins using the “enricher ()” function from the cluster Profiler package to identify overrepresented Gene Ontology (GO) terms. GO term annotations for *Solanum tuberosum* proteins were obtained from plant Ensembl Biomart using the biomaRt package. The whole proteome of *S. tuberosum* was used as the background “universe” for the network analysis. The 20 most significantly enriched pathways in each GO category were used for further analysis. Proteins involved in each of the selected pathways are presented in the table with their relative enrichment in log_2_ fold change (Peel_ratio) and the adjusted *p* value (Peel_p.adj).

**Supplementary Table 4. Potato root proteins enriched in network analysis**

Network analysis was performed on differentially expressed proteins using the “enricher ()” function from the cluster Profiler package to identify overrepresented Gene Ontology (GO) terms. GO term annotations for *Solanum tuberosum* proteins were obtained from plant Ensembl Biomart using the biomaRt package. The whole proteome of *S. tuberosum* was used as the background “universe” for the network analysis. The 20 most significantly enriched pathways in each GO category were used for further analysis. Proteins involved in each of the selected pathways are presented in the table with their relative expression in log_2_ fold change (Root_ratio) and the adjusted *p* value (Root_p.adj).

## Supplementary Figures

**Supplementary Figure 1.** **Differentially expressed and commonly shared proteins in peel and root derived EVs.**

(A) Enriched Proteins: Enriched proteins (log2 fold change > 2) in peel and root derived EVs. (B) Depleted Proteins: Depleted proteins (log2 fold change < -2) in peel and root derived EVs. (C) Unchanged Proteins: Proteins with no change in abundance (log2 fold change between 2 and -2) in peel and root derived EVs. The Euler diagrams highlight the percentage of proteins falling into each category.
